# Salivary volatilome profiling in pediatric eosinophilic esophagitis: a pilot study on a non-invasive approach in clinical practice

**DOI:** 10.1093/lifemedi/lnag012

**Published:** 2026-04-07

**Authors:** Rosamaria Capuano, Carla Petrella, Christian Barbato, Giulia D’Arcangelo, Giusy Russo, Alexandro Catini, Antonio Minni, Corrado Di Natale, Salvatore Oliva

**Affiliations:** Department of Electronic Engineering, and Interdepartmental Centre for Volatilomics “A. D’Amico”, University of Rome Tor Vergata, Rome 00133, Italy; Institute of Biochemistry and Cell Biology (IBBC), National Research Council (CNR), Sapienza University of Rome, Rome 00161, Italy; Institute of Biochemistry and Cell Biology (IBBC), National Research Council (CNR), Sapienza University of Rome, Rome 00161, Italy; Pediatric Gastroenterology and Liver Unit, Maternal and Child Health Department, Sapienza University of Rome, Rome 00161, Italy; Pediatric Gastroenterology, Hepatology and Cystic Fibrosis Unit, Fondazione IRCCS Cà Granda, Ospedale Maggiore Policlinico di Milano, Milan 20122, Italy; Pediatric Gastroenterology and Liver Unit, Maternal and Child Health Department, Sapienza University of Rome, Rome 00161, Italy; Department of Electronic Engineering, and Interdepartmental Centre for Volatilomics “A. D’Amico”, University of Rome Tor Vergata, Rome 00133, Italy; Department of Sensory Organs, Sapienza University of Rome, Rome 00161, Italy; Division of Otolaryngology-Head and Neck Surgery, San Camillo de Lellis Hospital, ASL Rieti-Sapienza University, Rieti 02100, Italy; Interdisciplinary Department of Well-being, Health and Environmental Sustainability (BeSSA), Sapienza University of Rome, Rieti 02100, Italy; Department of Electronic Engineering, and Interdepartmental Centre for Volatilomics “A. D’Amico”, University of Rome Tor Vergata, Rome 00133, Italy; Centre for Interdisciplinary Research (CIDR), SRM University-AP, Amaravati, Andhra Pradesh 522502, India; Pediatric Gastroenterology and Liver Unit, Maternal and Child Health Department, Sapienza University of Rome, Rome 00161, Italy

**Keywords:** volatile organic compounds, eosinophilic esophagitis, saliva, non-invasive monitoring, children

## Abstract

Eosinophilic esophagitis (EoE) is a chronic immune disease requiring repeated endoscopies for diagnosis and monitoring in children. Saliva represents a promising non-invasive biofluid, and volatile organic compounds (VOCs) may indicate disease presence and activity. This study aimed to examine the VOCs profile in saliva samples from children with EoE and to compare it with other gastrointestinal (GI) conditions and healthy controls. Thirty-five samples from children with EoE (including 13 active and 22 non-active cases), 19 from children with other GI conditions, and 46 from healthy controls were analyzed. Gas chromatography–ion mobility spectrometry (GC–IMS) identified 63 distinct VOC signal areas. The abundance of 16 of them was found significantly different (*P *< 0.01) in EoE vs. controls, EoE vs. other GI conditions, and active vs. non-active EoE. Among them, *cis*-3-hexen-1-ol and 2-phenylethanol show a ubiquitous capability to discriminate EoE against different populations. Linear discriminant analysis (LDA) of the panel of 16 VOCs achieved 83.3% accuracy in classifying EoE vs. healthy controls, 81.2% accuracy in distinguishing EoE from GI controls, and 80.0% accuracy in classifying active vs. non-active EoE. Salivary VOC profiling enables accurate discrimination of pediatric EoE from controls and stratification by disease activity. This non-invasive approach holds promise as a diagnostic and monitoring tool in clinical practice, especially in children.

## Introduction

Eosinophilic esophagitis (EoE) is a chronic, immune-mediated inflammatory disease of the esophagus characterized by eosinophil-predominant inflammation and related esophageal dysfunction [1]. Once considered rare, EoE has become a leading cause of upper gastrointestinal (GI) morbidity in children and adolescents. Recent epidemiologic studies estimate a prevalence of about 0.5–1 per 1000 (about 34 per 100,000) children, with an incidence around 5–10 cases per 100,000 per year [2, 3]. The incidence and recognition of EoE have risen markedly over the past two decades, paralleling the increasing prevalence of allergic disorders in developed countries. EoE often coexists with atopic conditions (up to 50%–70% of pediatric cases have asthma, eczema, or allergic rhinitis) and is considered part of the allergic march in many patients [4–6].

The current gold standard for EoE diagnosis is invasive endoscopy with esophageal biopsies, demonstrating ≥15 eosinophils per high-power field (Eos/HPF) on histological examination [1, 7]. While endoscopy is essential for initial diagnosis and assessment of mucosal healing, it is costly, requires general anesthesia in children, and carries the risks and burden of repeated procedures [8, 9]. Children with EoE frequently undergo multiple endoscopies to evaluate treatment response (e.g. dietary elimination or pharmacological therapy) and to monitor disease remission or relapse. This underscores the need for non-invasive biomarkers that could complement or partially replace endoscopic histology for diagnosing and monitoring EoE [10]. Candidate less-invasive approaches under investigation include the esophageal string test or sponge cytology devices for sampling esophageal content and analysis of exhaled breath condensates [10–12].

In recent years, saliva has emerged as an attractive diagnostic fluid in EoE and other GI diseases, as it can be obtained easily and repeatedly without discomfort. Saliva contains a complex mixture of host-derived molecules and microbiome products, reflecting both local oral/esophageal and systemic physiology. Prior studies indicate that salivary biomarkers may hold promise in EoE. For example, salivary microRNAs were recently shown to distinguish pediatric EoE patients from controls with about 70% sensitivity and specificity [11]. Previous studies using untargeted metabolomics of saliva have identified distinct metabolic signatures in children with EoE, including reduced levels of anti-inflammatory purine metabolites (adenosine, inosine) in active EoE compared to controls. These findings suggest that EoE pathophysiology produces measurable changes in saliva composition [13]. However, no studies have yet explored the salivary volatile metabolite profile (volatilome) in EoE. Volatile organic compounds (VOCs) are small molecules with high vapor pressures that can be released by biological fluids and detected as gases. The human “volatilome” reflects VOCs from both endogenous metabolic processes and host–microbe interactions [14]. Disease-specific VOC patterns have been identified in exhaled breath, skin emanations, urine, and feces, offering a potential diagnostic tool for various inflammatory and metabolic conditions.

A range of analytical tools is available for volatilomic studies. Among them, ion mobility spectrometry (IMS), particularly when integrated with gas chromatography (GC–IMS) offers several advantages with respect to other techniques, as it can detect very low concentration of VOCs in a few minutes [15]. On the other hand, GC–IMS is characterized by a lower resolution in terms of VOC identification. Due to these characteristics, GC–IMS is particularly suitable for untargeted volatilomics, allowing for rapid and sensitive VOC fingerprinting.

GC–IMS has successfully distinguished GI conditions, including differentiating patients with celiac disease (CD) from healthy controls and those with refractory CD [16]. Given these precedents, we hypothesized that salivary VOC profiles in pediatric EoE might be altered and could serve as non-invasive biomarkers of disease presence and activity.

To test this hypothesis, we performed salivary volatilome profiling in children with EoE and compared them to non-EoE controls (including healthy children and those with other GI diseases). We used GC–IMS for VOC analysis and applied multivariate statistical modeling to determine whether salivary VOC patterns could differentiate EoE, and if specific VOCs correlated with active vs. non-active disease.

## Results

### Subjects

The cohort of patients included 35 children with EoE (13 active and 22 non-active), 19 with other GI conditions (CD and *H. pylori* (HP) infection), and 46 healthy controls. Table 1 summarizes the baseline characteristics of patients in the three groups.

**Table 1.** Baseline characteristics of children included in the study.

Characteristics of the population	EoE (*n *= 35)	CD (*n *= 14)	HP gastritis (*n *= 5)	Healthy controls (*n *= 46)
**Age, median (IQR)**	14 (9–17)	10.5 (6.7–13)	13 (8.5–15)	11 (8–14.5)
**Disease duration, median (IQR)**	36 (19–75)			
**Sex M, *N* (%)**	27 (77)	7 (50)	3 (60)	17 (37)
**Eosinophil count (× 10^3^/mL), median (IQR)**	0.31 (0.2–0.53)	0.13 (0.11–0.48)	0.4 (0.1–1.7)	0.16 (0.14–0.26)
**CRP (mg/dL), median (IQR)**	0.07 (0.06–0.1)	0.06 (0.06–0.23)	0.06 (0.06–1.6)	0.06 (0.06–0.1)
**ESR (mm/h), median (IQR)**	10 (4–17)	6.5 (2.7–12)	15 (4–37)	7 (4–16)
**Endoscopically active (EREFS ≥ 3), *n* (%)**	7 (20)			
**Histologically active (> 15 Eos/HPF)**	13 (37)			
**Therapy at the time of EGDS, *n* (%)**				
**PPI**	13 (37)			
**Oral viscous Budesonide**	18 (51.4)			
**Budesonide dispersable tablet**	5 (14)			
**Symptoms at the time of EGDS, *n* (%)**				
**Food impaction**	6 (17)		0	
**Epigastric pain**	5 (14)	1 (7)	3 (60)	11 (24)
**Heartburn**	8 (23)	/	2 (40)	10 (22)
**Dysphagia**	6 (17)	1 (7)	0	7 (15)

Abbreviations: CD: celiac disease; CRP: C-reactive protein; EGDS: esophagogastroduodenoscopy; EoE: eosinophilic esophagitis; Eos: eosinophils; ESR: erythrocyte sedimentation rate; HP: helicobacter pylori; HPF: high power field; IQR: interquartile range; PPI: proton pump inhibitors.

### VOC detection and identification

Saliva samples from 100 pediatric participants were analyzed using GC–IMS to evaluate the potential of salivary VOC profiling in EoE. Figure S1 shows an example of the typical result of GC–IMS analysis. Instrumental signals are organized in a matrix where 1D represents the elution time along the chromatographic column and the other dimension the time-of-flight in the ion mobility detector. Most of the matrix is empty; the highlighted area shows the detection of VOCs, and the integral of the signal in the area is a measure of the abundance of the compound.

**Figure 1.**
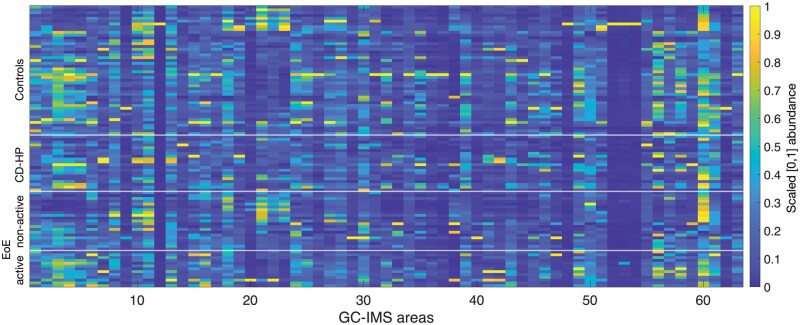
Heatmap of min–max rescaled VOCs abundance in all samples.

The analysis of the collected GC–IMS data identified 63 distinct VOC signal areas across all samples. A heatmap displaying the min–max normalized intensity of the 63 VOCs is shown in Fig. 1. The figure reveals marked interindividual variability in VOC composition; however, visual inspection alone was insufficient to reliably differentiate the four clinical groups.

To investigate the diagnostic potential of VOCs, three binary classification analyses were conducted. These compared individuals with EoE to healthy controls, EoE to CD–HP patients, and active EoE to non-active EoE cases. The Kruskal–Wallis test was employed, in each comparison, to identify VOCs that showed statistically significant differences in abundance. The resulting *P*-values are shown in Fig. S2.

**Figure 2.**
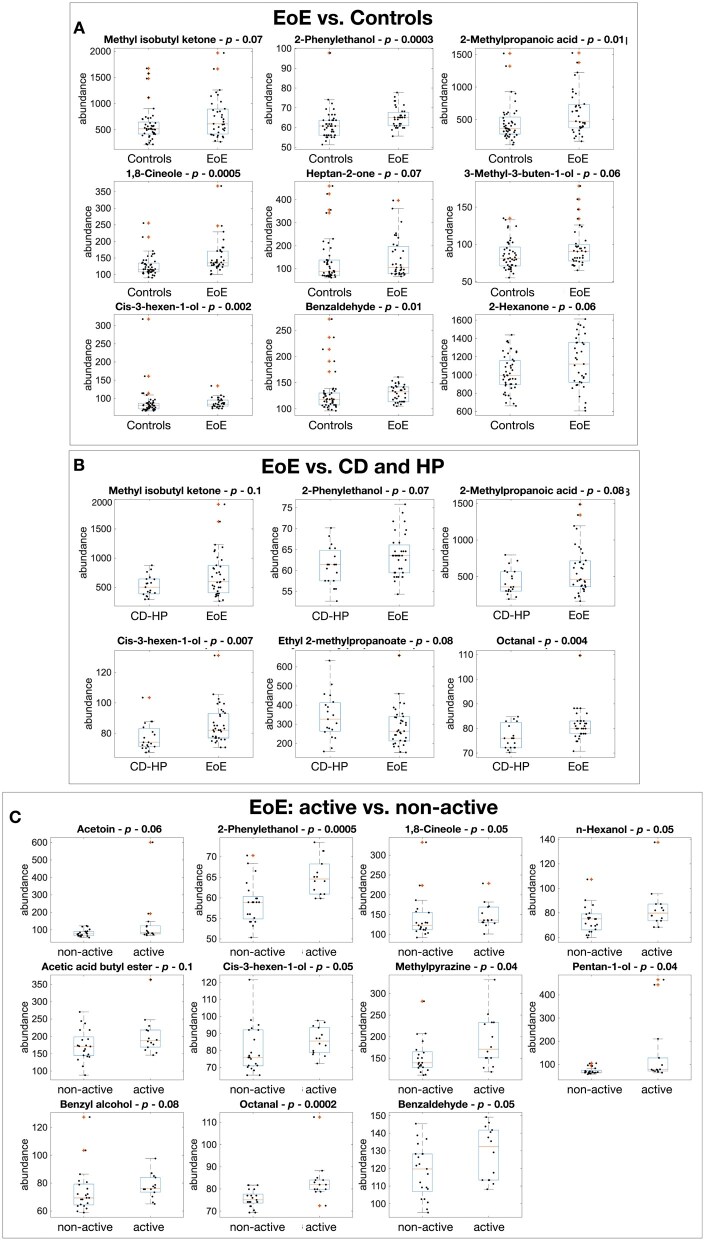
The distribution of the abundance on the compounds in Table 2 are compared as boxplots. (A) EoE vs. healthy controls. (B) EoE vs. CD and HP. (C) Active vs. non-active EoE. Boxplots are based on a normal distribution assumption. In each box, the central mark indicates the median, and the bottom and top edges of the box indicate the 25th and 75th percentiles, respectively. The whiskers extend to the most extreme data points not considered outliers, and the outliers are plotted individually using a red “+” marker symbol.

A subset of compounds demonstrating significant differences in the three classifications is detailed in Table 2. The compounds span a range of chemical classes, including organic acids, alcohols, aldehydes, alkanes, ethers, ketones, and pyrazines. Many of them are recognized components of the human volatilome and have previously been associated, in other biological matrices, with cancers and inflammatory or immune-mediated processes [14]. Figure 2 shows the distribution of abundances of the selected VOCs across the different binary classifications. Although the Kruskal–Wallis test revealed significant differences in VOC abundances, the conspicuous overlap between groups limits the diagnostic property of individual variables quantified by the AUROC (Fig. S3) [36].

**Table 2.** List of the most meaningful VOCs in each binary classification.^a^

VOC	EoE vs. controls	EoE vs. CD and HP	Active vs. non active EoE	Biological matrices	Associated disease	Ref.
***Cis*-3-hexen-1-ol**	*P *< 0.002	*P *< 0.007	*P *< 0.05			
**1,8-Cineole**	*P *< 0.0005		*P *< 0.05	Plasma, urine	Inflammation	[17–19]
**2-Hexanone**	*P *< 0.06			Breath	Asthma	[20]
**2-Methylpropanoic acid**	*P *< 0.01	*P *< 0.08		Saliva	Periodontitis	[21, 22]
**2-Phenylethanol**	*P *< 0.0003	*P *< 0.07	*P *< 0.0005	Feces	Ulcerative colitis	[23]
**3-Methyl-3-buten-1-ol**	*P *< 0.06			Breath	Emphysema	[24]
**Acetoin**			*P *< 0.06	Saliva	Periodontitis	[22]
**Acetic acid butyl ester**			*P *< 0.1			
**Benzaldehyde**	*P *< 0.01		*P *< 0.05	Urines	Kidney cancer; Idiopathic pulmonary fibrosis	[25, 26]
				Breath		
**Benzyl alcohol**			*P *< 0.08	Breath	Hypoxia	[27]
**Ethyl 2-methylpropanoate**		*P *< 0.08		Feces	Autism	[28]
**Heptan-2-one**					Ulcerative colitis	[29–32]
	*P* < 0.07			Saliva	CD	
					Oral cancer	
**Methyl isobutyl ketone**	*P *< 0.07	*P *< 0.1		Feces	GI disease	[23]
**Methylpyrazine**			*P *< 0.04	Urines	Lymphoma	[33]
***n*-Hexanol**			*P *< 0.05	Breath	Colon cancer	[34]
**Octanal**		*P *< 0.004	*P *< 0.0002	Breath	COVID-19	[32]
**Pentan-1-ol**			*P *< 0.04	Breath	CD	[35]

a The corresponding *P*-value is calculated from Kruskal–Wallis rank test. Previously detections in human volatilome are annotated in the table.

Abbreviations: CD: celiac disease; EoE: eosinophilic esophagitis; HP: helicobacter pylori; VOC: volatile organic compound.

Despite the limited properties of individual VOCs, their pattern, once properly analyzed with multivariate analysis methods, may reveal features exceeding those exhibited by univariate analysis [37]. For this scope, the patterns of VOCs were processed with machine learning-based algorithms, such as principal component analysis (PCA) and linear discriminant analysis (LDA).

**Figure 3.**
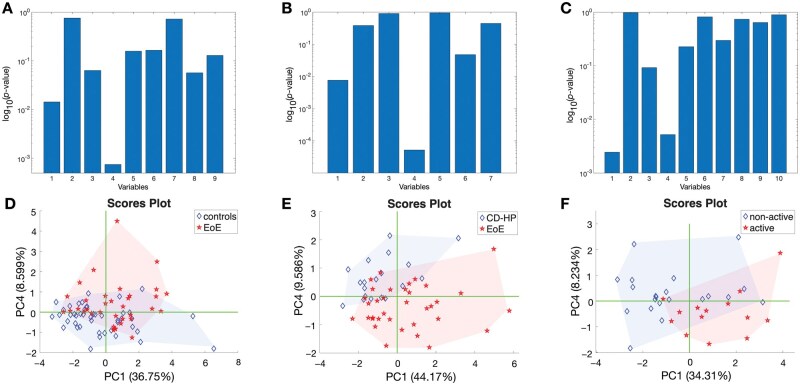
Results of PCA of datasets selected for each binary classification target. Results of Kruskal–Wallis rank test of principal components respect to EoE from healthy controls (A), EoE from CD and HP (B), and active and non-active EoE (C). Scores plot of the best discriminating principal components in case of EoE from controls (D), EoE from CD and HP (E), and active and non-active EoE (F).

PCA, a widely used method for dimensionality reduction, transforms the original variables into uncorrelated principal components while preserving as much variance as possible. The results of PCA are shown in Fig. 3. The statistical significance of the principal components in discriminating between classes was assessed using the Kruskal–Wallis test, with results displayed in Fig. 3A–C. The PCA results for the EoE vs. healthy controls and EoE vs. CD–HP comparisons were similar. This similarity suggests that CD–HP patients form a heterogeneous group whose internal differences do not enable them to form a specific group distinct from the controls. In both comparisons, the first principal component, which captures the greatest proportion of variance, achieved a *P*-value < 0.01. Interestingly, the fourth principal component was the most discriminative, with *P*-values below 0.001 and 0.0001 for EoE vs. controls and EoE vs. CD–HP, respectively. A slightly different pattern emerged in the comparison between active and non-active EoE. In this case as well, the first and fourth components are still those more statistically meaningful, both with *P*-values < 0.01.

The plot of the VOC patterns in the plane of the first and fourth principal components offers a visual representation of the discrimination properties (Figs. 3D–F). The plots are still characterized by a nonnegligible overlap of groups. However, in each case, the 2D PCA plots explained < 50% of the total data variance. Thus, much of the dataset’s information that lies outside the plots might potentially encode either additional information about EoE or biological patterns that may be related to further characteristics of the samples.

To gain a more robust understanding of the discriminatory power of VOC profiles, LDA was employed. This is a supervised classification algorithm aimed at determining a discriminant function, obtained as a linear combination of the input variables, that optimally separates the classes. For each of the three binary comparisons, the dataset was randomly partitioned into training and test subsets. Because classification performance can be sensitive to how data are split, LDA was repeated 100 times using different random partitions. At each step, an LDA model was trained and validated, and the performances were evaluated using a set of classification metrics: true positive rate, true negative rate, overall accuracy, and the area under the receiver operating characteristic curve (AUROC). The model with the classification metrics closest to the average across all iterations was selected as the representative model.

**Figure 4.**
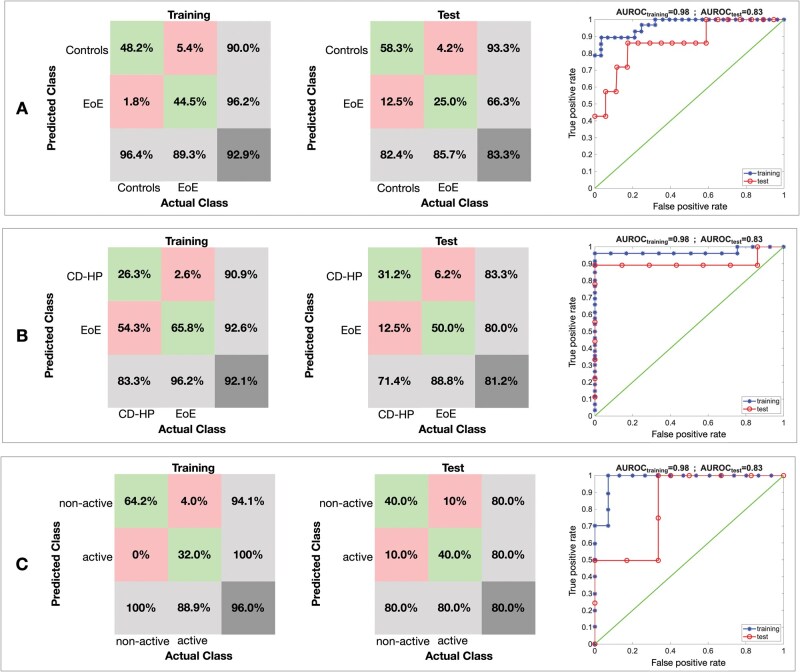
Performance of the classification models. (A) EoE vs. healthy controls. (B) EoE vs. CD and HP. (C) Active vs. non-active EoE. For each model the confusion matrices in training and test and the ROC curves in training and test are shown. The areas under the ROCs are printed in the header of ROC curve plots.

Figure 4 shows the confusion matrices and the receiver operating characteristic (ROC) curves for the representative LDA models. Performance metrics, including accuracy, sensitivity, specificity, and AUROC are summarized in Table 3; for true negative and positive rates, the interval of confidence has been estimated as the standard deviation over the 100 random dataset partitions. Results indicate strong classification performance in all comparisons. Specifically, for the EoE vs. healthy controls analysis, the model achieved an accuracy of 83.3% and an AUROC of 0.86. In the comparison between the EoE and CD–HP groups, accuracy was 81.2%, with an AUROC of 0.90. Finally, for distinguishing active from non-active EoE, the model reached an accuracy of 80.0% and an AUROC of 0.83.

**Table 3.** Performance metrics of the optimal classifiers for each binary classification.^a^

		Accuracy (%)	True positive rate (%)	True negative rate (%)	AUROC
**EoE vs controls**	Training	92.9	89.3 ± 10	96.4 ± 11	0.96
	Test	83.3	85.7 ± 17	82.4 ± 18	0.86
**EoE vs CD–HP**	Training	92.1	96.2 ± 10	83.3 ± 11	0.97
	Test	81.2	88.8 ± 17	71.4 ± 18	0.90
**EoE active vs non-active**	Training	96.0	88.9 ± 10	100 ± 11	0.98
	Test	80.0	80.0 ± 17	80.0 ± 18	0.83

a The interval of confidence of true negative and positive rates is calculated as the standard deviation over the 100 random dataset partitions.

## Discussion

**Figure 5.**
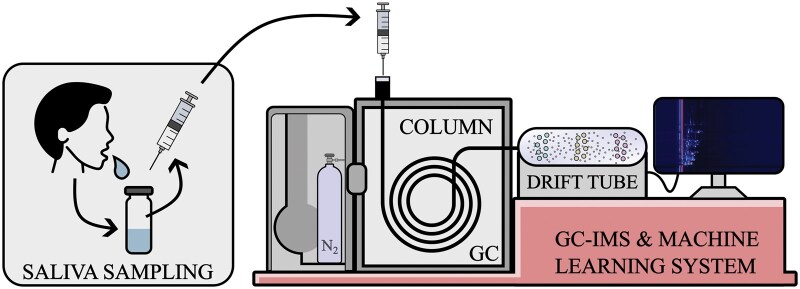
Schematic illustration of the experimental work. The headspace of saliva samples, collected from patients, are analyzed with GC–IMS and properly analyzed by machine learning algorithms to distinguish EoE respect to controls and CD or HP, and active from non-active forms of EoE.

This study is the first to investigate the relationship between salivary VOCs and EoE. Using GC–IMS, we characterized the salivary volatilome and applied statistical and machine learning approaches to distinguish patients with EoE from healthy controls, as well as from patients with CD and HP infection (Fig. 5). In addition, we tested the hypothesis that the salivary volatilome reflects disease activity, enabling discrimination between active and inactive stages of EoE.

Analysis of GC–IMS data selected a set of putatively identified potential VOCs specifically linked to EoE. The set contains representatives of various chemical families, and many of them have been previously detected in diverse human biofluids, where they were found associated with diseases, as noted in Table 2. For example, 2-hexanone, 3-methyl-3-buten-1-ol, benzyl alcohol, *n*-hexanol, octanal, and pentan-1-ol have been detected in breath; methyl isobutyl ketone, 2-phenylethanol, and ethyl 2-methylpropanoate in feces; benzaldehyde in both urine and breath; and methylpyrazine in urine [38]. Two compounds, 2-methylpropanoic acid (the short-chain fatty acid, iso-butyric acid) and acetoin have previously been identified in saliva, where they were correlated with gastroesophageal reflux disease and periodontitis, respectively [21–23]. Conversely, heptan-2-one was found in saliva and feces both in normal conditions [39] and in feces of children with CD [29] or ulcerative colitis [30, 31]. The limited correspondence with previous findings in saliva might likely reflect the scarcity of investigations on the salivary volatilome in EoE [13, 40].

Some VOCs, such as 1,8-cineole, methylpyrazine, and benzaldehyde, are likely of exogenous origin, possibly introduced through diet or environmental exposure [39]. However, their concentrations in saliva may still be modulated by the pathophysiology of EoE, suggesting that even exogenous compounds could have diagnostic utility [41]. Other VOCs, including 2-phenylethanol [42], benzaldehyde [43, 44], and *cis*-3-hexen-1-ol [45], are known products of microbial metabolism or lipid peroxidation and have been associated with inflammatory processes [46, 47, 48]. Furthermore, *cis*-3-hexen-1-ol, which functions as an insect attractant, plant metabolite, and air freshener [49], has not been associated with human metabolism or disease. The association of 2-phenylethanol and *cis*-3-hexen-1-ol with EoE is evidenced by the fact that these two molecules can discriminate EoE with respect to both controls and the mixed group of CD and HP. Furthermore, the abundance of these compounds is modulated by the active stage of the disease. Consequently, it is of utmost interest to further investigate these volatile compounds to determine their origin and connection with EoE.

The present results also show that 3-methyl-3-buten-1-ol and heptan-2-one were more abundant in individuals with EoE compared to healthy controls, but their concentration does not capture either the differences between EoE and other GI diseases nor between active and non-active disease states. Thus, they might be considered as general markers of GI inflammation rather than EoE-specific indicators. Finally, a subset of VOCs, including benzaldehyde, *n*-hexanol, pentan-1-ol, and methylpyrazine, being statistically different between active and non-active forms of EoE, suggests their potential utility for monitoring disease activity and progression.

However, the large overlap of signals in all the binary classifications indicated that the diagnostic properties of individual compounds are limited. Thus, rather than investigating single compounds, the patterns of the VOCs have been considered.

The diagnostic capabilities of VOC patterns were evaluated using an LDA classification algorithm. The results demonstrate good performance, with accuracy in tests above 80% and AUROC values exceeding 0.83 across all binary comparisons. These findings provide evidence that saliva can be a valuable diagnostic biofluid, at least for pediatric EoE.

The results are coherent with recent findings reported by Hiremath et al. [50] that recently applied untargeted liquid chromatography–mass spectrometry (LC–MS)-based metabolomics to saliva samples and identified over 400 significantly altered non-volatile metabolites in EoE patients compared to controls, including reduced levels of anti-inflammatory purines such as adenosine and deoxyadenosine. Another study demonstrated significant alterations in the salivary microbiome of children with EoE, including increased abundance of *Haemophilus* species correlating with disease activity [51]. Since many VOCs are microbial byproducts, their presence in saliva may reflect the inflammatory–microbial interface that is central to EoE [45, 51].

The approach of metabolomics addresses a critical clinical need for minimally invasive tools in EoE management. Dellon et al. [52] emphasized that while endoscopy with biopsy remains the diagnostic gold standard, its invasiveness limits its utility for frequent monitoring, particularly in pediatric populations [7]. They called for new diagnostic tools that are accurate, patient-friendly, and suitable for use in outpatient or home settings. In this context, GC–IMS–based VOC profiling offers a promising platform: it is rapid, label-free, requires minimal sample preparation, and can deliver real-time results with limited infrastructure.

Although our results clearly demonstrate that salivary VOCs can reliably identify disease presence and activity in a pediatric cohort, some limitations must be acknowledged. First, compound identification via GC–IMS remains putative without confirmatory validation using complementary analytical techniques. Second, as usual in cross-sectional studies, the influence of individual variability over time is not considered. Third, although repeated random sampling was employed to enhance robustness, external validation in larger, multicenter, and prospective cohorts is essential to assess the clinical validity of the results.

In conclusion, this paper suggests that the analysis of salivary VOCs using GC–IMS is a feasible and non-invasive method for identifying EoE and assessing disease stage. Future studies should aim to validate these findings in larger cohorts and to integrate volatilomics with non-volatile metabolites detected in saliva [14] to define a comprehensive molecular profile of the diseases that can result in a clinical tool for screening, diagnosis, and longitudinal monitoring, particularly in pediatric populations.

## Research limitations

This study provides a proof-of-concept demonstration that salivary volatile compound analysis carries diagnostic potential for EoE. Nonetheless, several limitations must be acknowledged.

First, the cohort was recruited at a single center, and all analyses were performed using a specific GC–IMS platform. As a result, potential influences related to local environmental exposures, dietary habits, and instrument-specific performance cannot be excluded. Future multicenter studies, ideally involving different analytical platforms, will be necessary to assess generalizability and robustness.

Second, methodological constraints must be considered. Compared with other biological matrices, the salivary volatilome remains relatively underexplored, and standardized protocols for collection, storage, and analytical workflows are not yet established. In this study, a basic and broadly applicable approach was intentionally adopted to demonstrate feasibility. However, further optimization of pre-analytical handling, sample conditioning, and instrumental settings will be essential to enhance sensitivity and improve the detection of disease-specific volatilomic signatures.

Together, these limitations highlight the need for larger, externally validated studies and methodological refinement. Nevertheless, the present findings provide a solid foundation for the development of saliva-based volatilomic tools for non-invasive EoE diagnosis and monitoring.

## Methods

### Research ethics

The study was conducted in accordance with the Declaration of Helsinki and approved by the Ethics Committee of the “Policlinico Umberto I” Hospital (protocol No.: 7812/24). All participants and/or their guardians gave written informed consent to participate in the study.

### Study design and population

This prospective, cross-sectional study was conducted at the Pediatric Gastroenterology and Hepatology Unit of Policlinico Umberto I in Rome, Maternal Child Health Department, Sapienza University of Rome, a tertiary pediatric gastroenterology center. Pediatric patients were recruited on the day of their scheduled endoscopy. Children aged 5–18 years were consecutively enrolled between 2023 and 2024 and assigned to one of three groups: EoE, GI disease controls, or healthy controls.

The EoE cohort included patients with an established diagnosis of EoE based on standardized pediatric guidelines, which required: (i) a compatible upper GI endoscopy with at least six biopsies from the esophagus (at least two samples from both upper and lower esophageal levels) and (ii) histologic confirmation of EoE with a peak eosinophil count of ≥ 15 Eos/HPF in esophageal biopsy specimens, in an appropriate clinical context, according to the European Society for Pediatric Gastroenterology, Hepatology and Nutrition (ESPGHAN) guidelines [1].

Within the EoE cohort, patients were further classified as active EoE (histologically active, defined by continued eosinophil density ≥ 15 Eos/HPF on current biopsy and/or persistent clinical symptoms) or non-active EoE (in histologic remission, < 15 Eos/HPF, with clinical improvement under therapy). All EoE patients were managed with standard therapies, including proton pump inhibitors (PPIs) and/or topical corticosteroids, as per clinical guidelines.

The disease control cohort consisted of children undergoing endoscopy for other conditions. Specifically, patients with CD (biopsy-confirmed villous atrophy with positive celiac serology) and those with *Helicobacter pylori* (HP) gastritis (biopsy-confirmed infection) were included. These conditions were chosen as inflammatory GI diseases unrelated to EoE to evaluate the specificity of any VOC findings.

The healthy control group included children without any known GI disease. Many healthy controls were asymptomatic siblings of patients or children undergoing minor procedures unrelated to the GI tract. Exclusion criteria for all groups included: (i) refusal to provide saliva samples, (ii) refusal to sign informed consent (by parents/legal guardians and/or the patient), and (iii) presence of other chronic intestinal or extraintestinal immune-mediated conditions requiring anti-inflammatory, steroid, or immunomodulator treatment.

For each participant, demographic information (age, sex) and clinical details were recorded. In the EoE group, disease duration, current treatments, and the presence of atopic comorbidities (such as asthma, allergic rhinitis, and eczema) were noted. Endoscopic and histologic findings were recorded at the time of diagnostic or follow-up endoscopy when saliva was collected.

### Saliva collection and handling

Unstimulated whole saliva was collected under standardized conditions. Participants were instructed to refrain from eating, drinking, or performing oral hygiene procedures for at least 1 h prior to sampling. Approximately 1.5 mL of saliva was collected from each participant into sterile tubes. The samples were immediately aliquoted into 20 mL headspace vials, sealed, and stored at −80°C until analysis and thawed only once prior to analysis. Before volatilomic profiling, saliva samples were thawed and allowed to equilibrate at 4°C for about 12 h. This process facilitated gradual thawing and preparation for headspace analysis.

### GC–IMS analysis

VOCs were analyzed using gas chromatography coupled with ion mobility spectrometry (GC–IMS) via the FlavourSpec^®^ system (G.A.S., Dortmund, Germany). The system was equipped with an HT2000H autosampler (HTA, Italy) and a 30 m × 0.53 mm I.D. × 1 µm MXT-5 column (Restek, USA). Saliva headspace samples were heated at 50°C for 10 min before automated injection (700 µL) using a 2.5 mL syringe at 70°C.

The GC operated at 45°C with a carrier gas flow starting at 5 mL/min, ramped to 50 mL/min over 10 min, and held constant thereafter. The IMS drift tube (5.3 cm) was maintained at 45°C and 2.7 kV, using nitrogen as both carrier and drift gas. Analysis was performed in positive ion mode.

### VOC detection and identification

Data acquisition and processing were carried out using VOCal and LAV software (G.A.S., Dortmund). VOC signal areas were identified and integrated using automatic peak detection across the drift time vs retention time plane. Putative identification was performed by comparing drift time and retention index values against the GC-IMS library (v0.1.3). The Kovats index was calculated using standard *n*-ketones (C4–C9) under the same GC–IMS conditions used for saliva analysis. The retention index (RI) of the considered standard compounds was calculated and compared with the RI of reference *n*-ketones. The correct alignment of GC–IMS data was validated by comparing the signals obtained from pure standards with those of the corresponding compounds detected in saliva samples. The standards used were pentan-1-ol, 2-hexanone, and heptan-2-one. Retention index and drift time (time of flight) values for both standards and samples are reported in Table S1. The differences between the values were < 0.1%, indicating optimal alignment of the GC–IMS chromatograms.

### Statistical and computational analysis

To identify VOCs that differed between groups, the Kruskal–Wallis rank test was applied to signal areas across three binary comparisons: EoE vs. healthy controls, EoE vs. CD–HP group, and active vs. non-active EoE.

PCA was employed to reduce data dimensionality and assess group clustering. PCA was performed on standardized data (zero mean, unit variance), and the most informative principal components were selected for visualizing group separation.

Supervised classification was performed using LDA. The model was iteratively trained and validated over 100 randomized data splits, with two-thirds of the dataset used for training and one-third for testing in each iteration. Classification performance was evaluated using metrics including accuracy, true positive rate, true negative rate, and area under the receiver operating characteristic curve (AUROC), with mean values and standard deviations calculated across all runs.

## Supplementary Material

lnag012_Supplementary_Data

## Data Availability

The data sources used for this study contain protected patient information and cannot be publicly shared. However, selected analysis code with patient-identifying information removed is available upon request by contacting the corresponding author.
