# Supplementary material for: Salivary volatilome profiling in pediatric eosinophilic esophagitis: a pilot study on a non-invasive approach in clinical practice
Source: Life Med. 2026 Apr 7;5(2):lnag012. doi: 10.1093/lifemedi/lnag012 (PMC13148378; doi:10.1093/lifemedi/lnag012)
Supplement: lnag012_Supplementary_Data [file lnag012_supplementary_data.docx]

**Salivary volatilome profiling in pediatric eosinophilic esophagitis: a pilot study on a non-invasive approach in clinical practice**

Rosamaria Capuano^1,#^, Carla Petrella^2,#^, Christian Barbato^2^, Giulia D’Arcangelo^3,4^, Giusy Russo^3^, Alexandro Catini^1^, Antonio Minni^5,6,7,*^, Corrado Di Natale^1,8,*,^ Salvatore Oliva^3,*^

^1^Department of Electronic Engineering, and Interdepartmental Centre for Volatilomics “A. D’Amico”, University of Rome Tor Vergata, Rome 00133, Italy

^2^Institute of Biochemistry and Cell Biology (IBBC), National Research Council (CNR), Sapienza University, Rome 00161, Italy

^3^Pediatric Gastroenterology and Liver Unit, Maternal and Child Health Department, Sapienza University of Rome, Rome 00161, Italy

^4^Pediatric Gastroenterology, Hepatology and Cystic Fibrosis Unit, Fondazione IRCCS Cà Granda, Ospedale Maggiore Policlinico di Milano, Milan 20122, Italy

^5^Department of Sensory Organs, Sapienza University of Rome, Rome 00161, Italy

^6^Division of Otolaryngology-Head and Neck Surgery, San Camillo de Lellis Hospital, ASL Rieti-Sapienza University, Viale Kennedy, Rieti 02100, Italy

^7^Interdisciplinary Department of Well-being, Health and Environmental Sustainability (BeSSA) Sapienza University of Rome, Rieti 02100, Italy

^8^Centre for Interdisciplinary Research (CIDR), SRM University-AP, Amaravati, Andhra Pradesh 522502, India

^#^These authors contributed equally to this work.

^*^Correspondence: salvatore.oliva@uniroma1.it (S.O.), dinatale@eln.uniroma2.it (C.N.), antonio.minni@uniroma1.it (A.M.)

**Supplementary Table S1.**

Drift time and retention index of pure standards and the same compounds in samples.

Drift time is give in arbitrary units respect to the Reactant Ion Peak (RIP).

|  |  |  |  |  |
| --- | --- | --- | --- | --- |
|  | Saliva samples | | Reference standard | |
| Compound | Drift time (a.u.) | Retention Index | Drift time (a.u.) | Retention Index |
| pentan-1-ol | 1.50875 | 766.2 | 1.5096629 | 765 |
| 2-Hexanone | 1.193 | 796.3 | 1.1897453 | 792 |
| Heptan-2-one | 1.64524 | 884.4 | 1.6339977 | 884.4 |


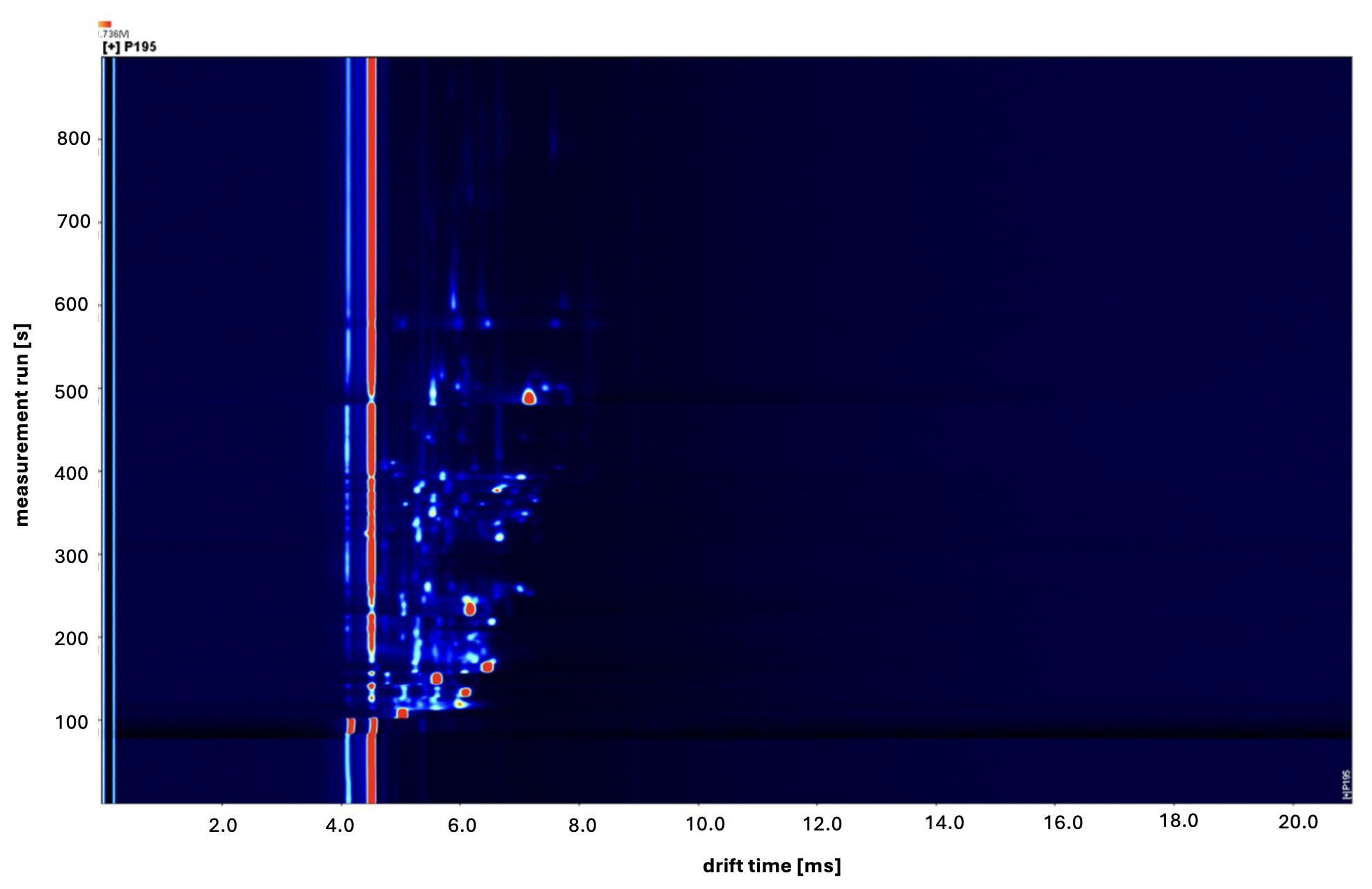


**Supplementary Figure S1.** Typical results of GC-IMS analysis. The intensity of signals is represented as a heat-map in the drift time (horizontal axis) vs. retention time (vertical axes) plane. Each area of the figure corresponds to a compound characterized by a retention and drift times. The integral of the signal in the area is a measure of the abundance of the corresponding VOC.

**Supplementary Figure S2.** Results of Kruskal–Wallis rank test of GC–IMS areas respect to the discrimination of the EoE patients vs. the healthy controls, the group of CD and HP, and between active and non-active EoE.

**Supplementary Figure S3.** Receiver Operating Characteristic curves of selected VOCs in the three binary classifications. The area under the ROC curve is indicated in the header of each plot.
